# Body Composition and Strength Symmetry of Kettlebell Sport Athletes

**DOI:** 10.3390/biology12030440

**Published:** 2023-03-12

**Authors:** Silvia Stagi, Gabriele Mulliri, Azzurra Doneddu, Giovanna Ghiani, Elisabetta Marini

**Affiliations:** 1 Department of Life and Environmental Sciences, Neuroscience and Anthropology Section, University of Cagliari, Cittadella di Monserrato, 09042 Cagliari, Italy; emarini@unica.it; 2 Department of Medical Sciences and Public Health, University of Cagliari, 09124 Cagliari, Italy; jabutele84@gmail.com (G.M.); didi-zazzy@hotmail.it (A.D.); giovanna.ghiani@tiscali.it (G.G.)

**Keywords:** segmental body composition, phase angle, *specific* BIVA, hand grip strength, symmetry

## Abstract

**Simple Summary:**

Segmental and total body composition, along with hand grip strength, are associated with performance, success, and health in athletes. Body composition and strength symmetry may be modulated by physical activity. *Specific* bioelectrical impedance vector analysis (BIVA) is an accurate, easy-to-use, and economical tool for evaluating body composition, suitable for sports applications. In this study, we evaluated hand grip strength, body composition, and body symmetry in a sample of elite athletes of both sexes practicing kettlebell sport (KBs), which is an increasingly practiced sport based on the repetition of jerk or snatch exercises realised using both upper limbs. The results suggest that kettlebell sport is associated with high muscle mass, and body composition and strength symmetry, especially in the upper body. As previously detected in other elite athletes, body symmetry could be related to the bilateral use of the limbs.

**Abstract:**

Kettlebell sport (KBs) is increasingly popular, but very few studies have been focused on this discipline. This research aims to investigate the effects of KBs on body composition, strength symmetry, and segmental body composition symmetry in a sample of Italian elite athletes. Data were collected from a sample of 16 athletes of both sexes (11 men and 5 women; 34.5 ± 9.0 years of age). Anthropometric (height, weight, arm, thigh, calf, and waist circumferences), hand grip strength, and total and segmental bioelectrical variables were taken. Body composition was analysed by using *specific* bioelectrical impedance vector analysis (BIVA). Paired *t*-tests and confidence ellipses were applied to analyse bilateral differences. Elite athletes of both sexes showed high values of phase angle, indicative of high body cell mass and quality and proxy of muscle mass. Hand grip strength and body composition were symmetrical, with the only exception of a higher %FM in the right leg (Zsp: t = 3.556; *p* = 0.003). In conclusion, this study suggests that KBs contributes to muscle mass improvement, body composition, and strength symmetry, especially in the upper body.

## 1. Introduction

The bilaterally paired structure of the human body is commonly associated with antisymmetry, which is the asymmetrical expression of a trait on the left and right side regardless of which side has more remarkable development [1]. Antisymmetry depends on genetic and environmental factors and is typically influenced by the differential use of body segments [2,3,4]. It can be observed in the different sizes, body composition, strength, and functionality of contralateral limbs and is widespread in the general population and among athletes [5]. In both sexes and through the life cycle, the dominant leg and arm (more frequently the right arm) show higher values of lean mass and strength [2,6], while the differences in the fat mass (FM) are generally less accentuated [6]. Among athletes, some disciplines, such as tennis, fencing, and soccer, are more commonly associated with differences between sides [7,8,9,10,11,12,13]. Indeed, the dominant upper or lower limbs of athletes practicing tennis [12,13] or soccer [10,11] have shown higher muscle mass and strength. Instead, other physical activities, such as walking, are associated with body symmetry [11]. 

In sports science, studies on asymmetry focused on physical capacity and strength more than on body composition and mainly aimed to analyse their relationship with performance or risk of injuries. Although asymmetry has been frequently considered to be related to adverse outcomes, recent reviews doubt the robustness of such associations and highlight the need for further research [5,14,15,16,17]. The literature results are still controversial because of the large variability of characters and phenomena under study, which include different outcomes (size, body composition, performance, injury risk), different body segments (anthropometric details, upper or lower limbs…), different kinds of relationships (causal or not), different types of asymmetry (antisymmetry or fluctuating asymmetry), and asymmetry indices. A major cause of variability is the heterogeneity among disciplines, that involve different motor tasks and training practices, thus resulting in different patterns of asymmetry.

Kettlebell sport (KBs, or girevoy sport) is a weightlifting discipline aiming to perform the highest number of repetitions of lifting the kettlebell or girya in ten minutes. This sport involves ballistic movements using both the left and right side of the body similarly: jerk exercises—performed with two kettlebells at the same time, lifted from the ground (long cycle) or the chest (jerk) to overhead—and snatches—performed swinging one kettlebell between the legs and lifting to the overhead position, using one arm at a time [18]. Although practiced for decades in many countries, the kettlebell sport was recognized in Russia in the 1990s [19], and the international federations (World Kettlebell Sport Federation, WKSF; International Gira Sport Federation, IGSF) started their activity in very recent years. Currently, KBs competitions include different sex and weight categories based on the type of exercise (jerk, snatch, and long cycle, or their different combinations) [20]. 

KBs differs from kettlebell training, which is the frequently practiced use of the kettlebell as a tool for physical workout. Indeed, the kettlebell has been used among Russian soldiers since the late 1800s [19] and is currently included in different types of physical training to increase strength and weightlifting capacity. Unlike kettlebell sport, which is based on the repetition of standardised exercises involving the same groups of muscles, kettlebell training is variable and is based on different movements and involving different muscles, depending on the kind and purpose of the training. Kettlebell training showed effectiveness in improving cardiovascular capacity [21,22] and muscle strength [23,24,25]. Due to safety during use [26] and potential therapeutic efficacy, KB training is suitable in clinical practice, physiotherapy interventions [27], and physical activity programs for the elderly. Among older individuals with sarcopenia, it has demonstrated efficacy in increasing grip strength [28].

The literature on KBs is less represented than that on kettlebell training. Previous studies on KBs have focused on improvements in the physical capacities of Ukrainian cadets [29] or on physiological indicators related to the competitive results of KBs athletes [30]. To our knowledge, only one study evaluated body composition (using anthropometry) in a sample of KBs athletes [18], whereas research on hand grip strength or symmetry in strength and body composition still must be addressed. However, their analysis can be useful in describing the physical peculiarities of athletes’ performances and in adding information on bilateral differences.

A variety of methods and instrumentations are available to evaluate body composition. Phase angle (PhA) and bioelectrical impedance vector analysis (BIVA) have proven to be accurate, inexpensive, and minimally invasive tools [31,32,33,34,35,36,37,38,39,40]. PhA is an indicator of the quantity and quality of cell membranes and is considered a marker of muscle quality and function [36,37,38]. It is also related to the distribution of body fluids, particularly of extracellular to intracellular water ratio (ECW/ICW; 33), and depends on age, height, fat-free mass (FFM) [37], muscle strength [39], and physical activity [35]. The *specific* BIVA approach [32], which analyses PhA along with vector length, has also shown accuracy in the evaluation of the relative content of fat mass both at the total [32] and the segmental level [40]. It is suitable for applications in the general population and various clinical and sports settings [32,33,34,35,41,42].

In order to improve our scant understanding of Kettlebell sport, the present work was aimed at evaluating total and segmental body composition and strength and their bilateral differences in a sample of Italian elite athletes of both sexes. Considering the peculiarities of the discipline and the mesolabile nature of the variables under study, we expect to detect morpho-functional symmetry.

## 2. Methods

### 2.1. The Sample 

The sample examined in this study is composed of 16 kettlebell sport athletes (11 men and 5 women), aged between 24 and 58 years, recruited from the Hanuman Training Center (Cagliari, Italy). Four athletes had the qualification of Master of Sport, and the others were candidates for qualification or competed at the regional or national level. The years of training were in mean 2.7 ± 1.2.

Participants were informed about the objectives and procedures of the study and signed their consent to participate. The Independent Ethical Committee of the A.O.U. of Cagliari (PG/2017/1700) approved the study.

Exclusion criteria were the presence of pathologies that might influence the measurements (e.g., significant cardiovascular diseases), metallic prostheses or pacemakers, and pregnancy.

### 2.2. Anthropometric Measurements

Height (cm, to the nearest 0.1 cm); weight (kg, to the nearest 0.5 kg); relaxed arm, waist, proximal thigh, and calf circumferences; and arm and leg lengths (cm, to the nearest 0.1 cm) were taken by an experienced operator following a reference protocol [43], using Seca (Hamburg, Germany) scale (model 761), tape (model 201) and stadiometer (model 217). BMI was calculated as weight/height^2^ (kg/m^2^). BMI and waist circumferences were evaluated based on the thresholds adopted by the WHO [44].

### 2.3. Hand Grip Strength

Hand grip strength was measured with a hydraulic dynamometer (Saehan Corporation, South Korea). Each participant was asked to stand, keeping the elbow flexed at 90°, holding the instrument and squeezing with the maximum possible force [45]. The measures were taken three times on each side, alternating the two sides to allow recovery between repetitions. The maximum value of the three repetitions was considered. Hand grip values were compared to the age- and gender-specific cut-offs of the general population [46] and to athletes practicing different sports [47]. 

### 2.4. Bioimpedance Analysis

Bioelectrical values (resistance, R, ohm; reactance, Xc, ohm) were taken for the total body and each body segment (arms, legs, right and left sides) with a single-frequency phase-sensitive bioimpedance device (50 kHz and 400 µA; BIA 101 Anniversary, Akern, Firenze, Italy). The device was calibrated before each measurement session with a circuit tester (R = 380 ohm, Xc = 47 ohm; 2% error). According to standard procedures, measurements were taken with the subject in a recumbent position and lying on a nonconductive surface [48]. Participants were asked to not drink or eat for at least three hours, to not exercise for at least eight hours, and to empty their bladders before the measurement. The electrodes used in this study were Biatrodes (Akern, Firenze, Italy). For the total body evaluation, two pairs of electrodes were placed on the right side of the body, precisely on the wrist and at the base of the second and third metacarpal and on the ankle and at the base of the second and third metatarsal [48]. For the arm, a pair of electrodes was placed at the level of the acromion and 5 cm below and on the hand (in the same position as the total body); for the leg, a pair of electrodes was placed at the level of the iliac crest and 5 cm below and in the foot (in the same position of the total body) [40]. Body composition was evaluated with *specific* BIVA [32]. R and Xc values were adjusted for a correction factor (A/L), where A is the cross-sectional area (C^2^/4π cm^2^) of the total body (calculated as A = 0.45 arm area + 0.10 waist area + 0.45 calf area) or the body segment and L is the height, or the length, of the body segment [32,40]). *Specific* vector length (Zsp) was calculated using the formula Zsp = (Rsp^2^ + Xcsp^2^)^0.5^ (ohm*cm), and phase angle (PhA) was calculated using the formula PhA = arctan Xc/R*180/π (°). According to *specific* BIVA, the vector length (Zsp) is positively associated with fat mass percentage (%FM), whereas the slope (PhA) is positively associated with body cell mass and body fluids distribution (ICW/ECW) and can be considered a proxy of muscle mass and quality. Following the method proposed by Piccoli et al. [31], the analysis of bioelectrical vectors was performed using tolerance and confidence ellipses. Tolerance ellipses represent the variability of bioelectrical vectors in a reference sample. In this study, a sample of Italo-Spanish young adults was used for comparison [49]. Confidence ellipses, representing the area where the average of the population falls with a probability of 95%, were used for intergroup comparisons.

### 2.5. Statistical Analysis

Descriptive statistics were calculated for anthropometrical, bioelectrical (total and segmental), and hand grip strength variables. Normality was checked using the Shapiro–Wilk test, and statistical tests were applied considering that all variables were normally distributed. Bioelectrical values of athletes were projected on the Cartesian plane and analysed through tolerance ellipses. Differences between the dominant and non-dominant arm and between the right and left leg were analyzed using a paired data Student’s *t*-test or confidence ellipses projected on the paired-data RXc plot along with Hotelling T^2^ test. Ellipses overlapping the origin of the paired data RXc plots indicate the lack of differences between bioelectric mean vectors, whereas ellipses in a different position indicate significant differences. The direction of the paired confidence ellipse shift on the graph can also furnish information on the prevailing contribution of resistance (if more shifted along the horizontal axis) or reactance (if more shifted along the vertical axis). The significance level was set at 0.05.

The software SPSS version 29 (SPSS Inc. Chicago, IL, USA), classic BIVA [50], and *specific* BIVA (www.specificbiva.com; accessed on 1 December 2022) were used for the statistical analysis.

## 3. Results

According to WHO cutoffs [44], the sample of athletes practicing KBs showed mean BMIs denoting normal weight in females and slight overweight in males; waist circumferences were within the normal values in both sexes (Table 1).

**Figure 1:**
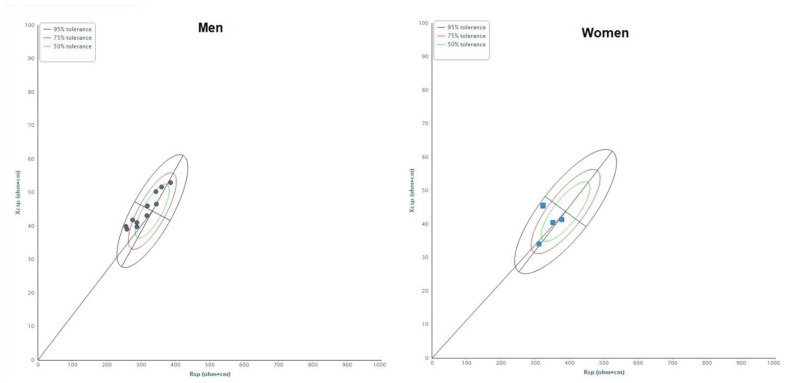
Distribution of bioelectrical vectors of athletes on the sex-specific tolerance ellipses. Vectors falling towards the upper pole indicate a tendency toward higher %FM, while those on the right side indicate a tendency toward greater cellular mass and ICW/ECW. Black dots: men; blue dots: women. Rsp: *specific* resistance; Xcsp: *specific* reactance.

Compared to the reference sample of young adults, KBs athletes of both sexes were characterised by high PhA values (more than 88% of the vectors falling on the left side of the ellipses), and KBs women by short vector lengths too (all cases in the lower part of the ellipse) (Figure 1). According to *specific* BIVA, this pattern indicates high body cell mass and ICW/ECW ratio in both sexes and low %FM in women.

**Figure 2:**
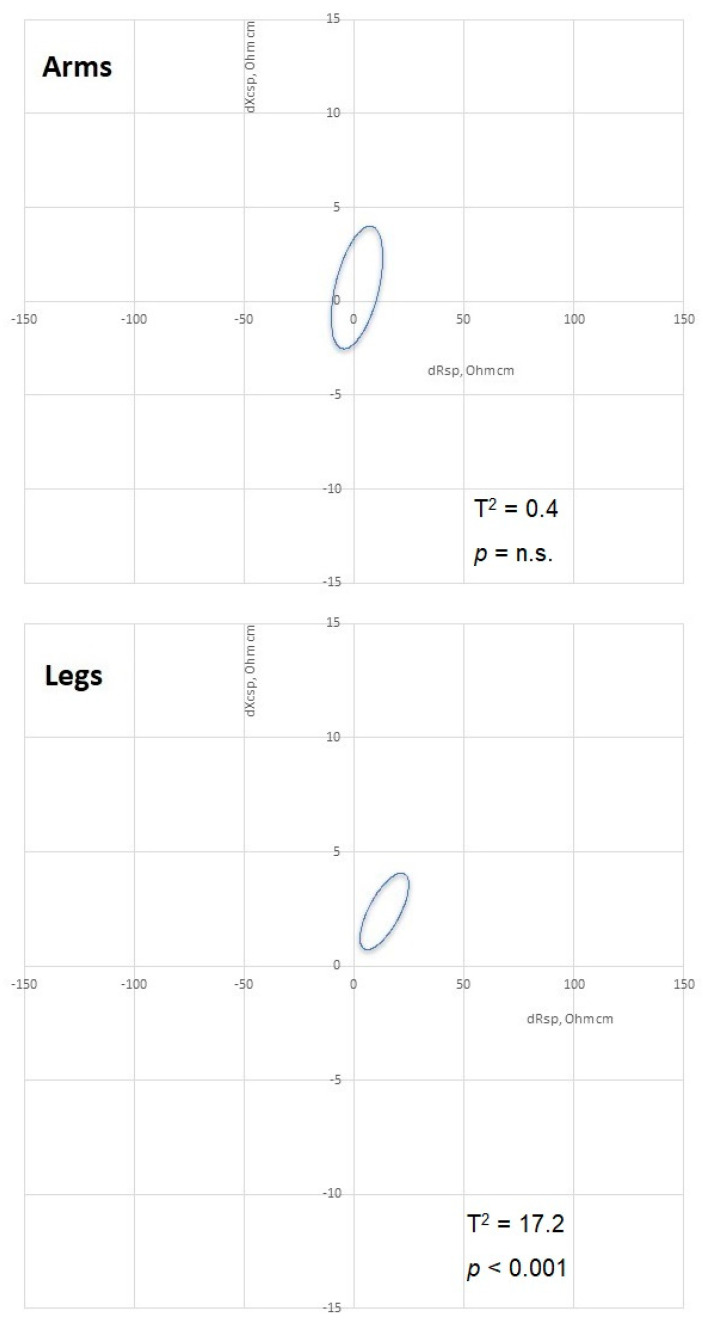
Paired data RXc graphs of the comparison between dominant and non-dominant arms and between right and left legs. Rsp: *specific* resistance; Xcsp: *specific* reactance.

The two sides showed few differences, limited to the leg. The arms were symmetrical in anthropometrical and bioelectrical measurements and hand grip strength, considering the differences between both the dominant and non-dominant arms (Figure 2) and the right and left sides (Table 2). The legs showed side differences in the calf circumference and Zsp, Xcsp, and Rsp, with higher values on the right side, whereas PhA values were similar on the two sides (Table 2). Confidence ellipses confirmed the pattern of side symmetry in the arm and asymmetry in the leg, with higher values on the right side (Figure 2). 

## 4. Discussion

This study aimed to analyze body composition and hand grip strength and their bilateral differences in athletes practicing kettlebell sport. KBs athletes of both sexes were characterised by total body bioelectrical values indicative of high cell mass and quality, mainly attributable to the skeletal muscle, compared to a reference sample of young adults [49]. Women also showed low levels of fat mass. A noticeable body symmetry in body composition values, especially in the upper body, and hand grip strength has been detected.

Compared to its cousin sport (weightlifting), KBs is considered an effective aerobic sport able to increase power and functionality parameters [18]. Therefore, it appears more closely related to endurance disciplines [18]. Accordingly, bioelectrical characteristics observed in the present research, particularly phase angle values, are near to those characterising endurance disciplines, such as cycling, marathon, pentathlon, sailing, skiing, rowing, and triathlon [35]. 

Previous studies applying *specific* BIVA to two different samples of young adults practicing various sports disciplines detected a vector distribution similar to that observed among kettlebell sport athletes [33,40]. In fact, both samples showed higher phase angle values and shorter vector length with respect to the same reference of young adults used in this study, especially among women. Stagi et al. [40] also applied *specific* BIVA at the segmental level. Compared to KBs athletes, the sample of active individuals analysed by Stagi et al. [40] was characterised by higher Zsp values at the arm level and higher PhA values at the leg level. The lower content of %FM in the arm of KBs athletes and the relatively low values of PhA in the leg can be attributed to the type of exercise, mainly interesting the upper limbs. 

Hand grip strength is considered a proxy of overall body strength [51]. In sports science and related applications, hand grip strength is used to furnish insights into sports performance and the effect of sports-specific movement patterns [47]. In fact, according to a recent review [47], elite athletes practicing different disciplines generally show higher hand grip strength than their sub-elite counterparts. Accordingly, athletes practicing KBs showed strength values similar to those observed by Fry et al. [52] in elite male weightlifter athletes. On the other side, compared to literature data on several sports (not including KBs), hand grip values of both male and female KBs athletes are in the average or lower range of variation [47]. Furthermore, KBs athletes showed hand grip values similar to those of the general population [46]. Indeed, as highlighted by Cronin et al. [47], the comparability of results on hand grip strength values can be hampered by the lack of standardization of procedures and instruments.

The relationship between exercise and body composition or strength symmetry has been investigated in some commonly practiced sports and age groups only. As in the general population, sports implying unilateral movements or kinetic asymmetries are generally associated with bilateral differences. Higher lean and bone mass and strength have been detected in the dominant arm of young elite tennis or handball players [9,53,54] or the dominant leg of ski racers and soccer or hockey players [10,11,55,56]. In contrast, disciplines implying a bilateral exercise show an opposite effect on segmental body composition, with a more symmetrical pattern between sides [11,55]. Also, the sport seems not to have a similar effect over life, as aged athletes practicing tai chi, running, and even tennis showed body symmetry at both the upper and lower body levels [42].

The symmetrical results observed in this study are probably due to KBs training and competition peculiarities. Indeed, unlike other disciplines involving unilateral or asymmetric motor tasks, KBs implies a bilateral symmetric use of the body, as the athletes stand and both upper limbs are used for lifting the gyria/kettlebell. In fact, both in jerk exercises, in which the athletes use simultaneously two kettlebells, and in snatch exercises, in which they use one arm at a time to lift one kettlebell (technically “hand change”), the use of left- and right-side muscles of the upper and lower body is guaranteed. Furthermore, the sequence of repeated ballistic movements in a short time probably emphasizes the effect on body composition and strength. These peculiarities are the key to the observed symmetry in strength and body composition, especially in the upper body. The leg showed a less symmetrical pattern, as the PhA indicated bilateral similarity of body cell mass, whereas %FM resulted slightly higher in the right leg. Similar results have been observed in long-term aged athletes [42], whereas in young elite tennis athletes and adult hockey players, the higher FM values were on the left side [12,56]. 

The major limitation of this study is the low sample size of athletes. In particular, the low number of women did not allow the analysis of sex differences. This is partly due to the peculiarities of KBs, a sport that still does not reach a great diffusion. Another limitation is that influencing factors related to lifestyle have not been analysed. On the other hand, the strengths of this work are that it is one of the very few studies on KBs and the first investigating body composition and strength symmetry in a sample of elite KBs athletes of both sexes.

## 5. Conclusions

In conclusion, elite kettlebell athletes showed body composition and strength symmetry, especially in the upper body, that could be related to the symmetrical exercise typical of this discipline. In the more common scenario of sports characterised by antisymmetry, KBs represents an interesting case study for analysing the relationships between symmetry and training or performance. 

*Specific* BIVA has proven to be an efficient and easy-to-apply method for studying total and segmental body composition, highlighting its usefulness in sports science applications. 

## 6. Practical Applications

The analysis of the total body and segmental body composition, hand grip strength, and their laterality can be useful to monitor athletes practicing kettlebell sport and for comparisons with athletes practicing other disciplines.

*Specific* BIVA is a reliable and simple procedure that can be used by researchers or trainers to easily investigate the body composition of athletes in different phases of their careers.

## Figures and Tables

**Table 1:** Descriptive statistics of anthropometrical and bioelectrical variables.

	Men (11)		Women (5)	
	Mean	SD	Mean	SD
Age (years)	36.29	9.63	30.41	6.70
Height (cm)	175.33	6.34	161.86	9.92
Weight (kg)	79.23	6.98	56.90	12.36
BMI (kg/m^2^)	25.80	2.30	21.55	3.51
Waist crf (cm)	83.01	5.32	67.24	5.39
Hand grip R (kg)	53.18	11.44	27.00	6.71
Hand grip L (kg)	49.82	9.21	27.40	8.47
Rsp (ohm·cm)	315.05	42.90	339.80	27.45
Xcsp (ohm·cm)	40.06	4.73	37.81	4.74
Zsp (ohm·cm)	317.59	43.12	341.93	27.31
PhA (°)	7.27	0.33	6.38	0.92

SD: standard deviation; BMI: body mass index; crf: circumference; Rsp: *specific* resistance; Xcsp: *specific* reactance; Zsp: *specific* vector length; PhA: phase angle.

**Table 2:** Laterality of strength, anthropometrical, and bioelectrical variables.

	Right Side				Left Side				Paired *t*-Test between Sides	
	Men		Women		Men		Women			
	Mean	SD	Mean	SD	Mean	SD	Mean	SD	t	*p*
Hand grip (kg)	53.18	11.44	27.00	6.71	49.82	9.21	27.40	8.47	1.742	0.102
Arm crf (cm)	32.35	1.64	26.14	2.02	32.33	1.92	26.42	2.55	−0.475	0.642
Thigh crf (cm)	59.61	3.68	54.64	5.96	59.84	3.64	55.04	5.93	−1.197	0.250
Calf crf (cm)	38.27	2.24	34.18	2.15	37.85	2.17	33.92	1.89	2.545	0.022
Rsp arm (ohm·cm)	233.85	30.09	243.56	25.64	232.95	27.08	254.07	33.98	−1.558	0.585
Xcsp arm (ohm·cm)	30.96	4.85	26.84	2.31	30.16	4.22	27.57	4.43	0.451	0.658
Zsp arm (ohm·cm)	235.91	30.27	245.06	25.41	234.94	26.92	255.57	34.17	−0.634	0.536
PhA arm (°)	7.57	0.89	6.36	0.98	7.45	1.31	6.20	0.58	0.488	0.633
Rsp leg (ohm·cm)	277.62	33.09	319.48	26.33	267.95	31.77	296.51	17.65	2.588	0.021
Xcsp leg (ohm·cm)	39.25	6.27	38.33	7.44	37.29	6.02	34.97	6.31	3.238	0.006
Zsp leg (ohm·cm)	280.39	33.59	321.82	26.70	270.54	32.25	298.61	17.77	3.556	0.003
PhA leg (°)	8.02	0.49	6.82	1.06	7.90	0.50	6.73	1.15	1.326	0.205

SD: standard deviation; BMI: body mass index; crf: circumference; Rsp: *specific* resistance; Xcsp: *specific* reactance; Zsp: *specific* vector length; PhA: phase angle.

## Data Availability

The data used in this study to support the findings are available from the corresponding author upon reasonable request.
